# Long-Term Survival of Individuals Born Small and Large for Gestational Age

**DOI:** 10.1371/journal.pone.0138594

**Published:** 2015-09-21

**Authors:** E. Christina M. Wennerström, Jacob Simonsen, Mads Melbye

**Affiliations:** 1 Statens Serum Institut, Department of Epidemiology Research, Copenhagen, Denmark; 2 Clinical Medicine, Copenhagen University, Copenhagen, Denmark; 3 Department of Medicine, Stanford School of Medicine, Stanford, CA, United States of America; The Ohio State Unversity, UNITED STATES

## Abstract

**Background:**

Little is known on long-term survival and causes of death among individuals born small or large for gestational age. This study investigates birth weight in relation to survival and causes of death over time.

**Methods:**

A national cohort of 1.7 million live-born singletons in Denmark was followed during 1979–2011, using the Danish Civil Registration System, the Medical Birth Registry and the Cause of Death Registry. Cox proportional hazards were estimated for the impact of small (SGA) and large (LGA) gestation weight and mortality overall, by age group and birth cohort.

**Results:**

Compared to normal weight children, SGA children were associated with increased risk of dying over time. Though most of the deaths occurred during the first year of life, the cumulative mortality risk was increased until 30 years of age. The hazard ratios [HR] for dying among SGA children ages <2 years were: 3.47 (95% CI, 3.30–3.64) and 1.06 (95% CI, 0.60–1.87) in 30 years and older. HR for dying among SGA adults (20–29 years) were: 1.20 (95% CI, 0.99–1.46) in years 1979–1982 and 1.61 (95% CI, 1.04–2.51) in years 1989–1994. The SGA born had increased risk of dying from infection, heart disease, respiratory disease, digestive disease, congenital malformation, perinatal conditions, and accidents, suicide, and homicide. Individuals born LGA were associated with decreased mortality risk, but with increased risk of dying from malignant neoplasm.

**Conclusions:**

Survival has improved independently of birth weight the past 30 years. However, children born SGA remain at significantly increased risk of dying up till they turn 30 years of age. Individuals born LGA have lower mortality risk but only in the first two years of life.

## Introduction

Several studies have investigated the association between birth weight and mortality and morbidity [1–4]. How birth weight is taken into account such as absolute birth weight, centile charts and other weight definitions has been a topic of debate and has also recently been evaluated [3]. Though birth weight is highly correlated with morbidity and mortality, it is not a true risk factor but rather the result of one or several causing events. The underlying mechanism of intrauterine growth is not fully understood but hypotheses gather around maternal metabolic vascular health during pregnancy and differences in placental implantation [5]. In the 1990s, David Barker formulated a hypothesis suggesting that events occurring during intrauterine life and in early infancy can influence the occurrence of diseases in adulthood. This theory proposes that undernutrition and other insult or adverse stimulus in utero and during infancy can permanently change the body's structure, physiology and metabolism, and the lasting or lifelong effects will depend on the period in the development or gestational week at which it occurs [6].

Over the past 40 years perinatal care has improved survival of preterm and low-birth-weight infants tremendously. For example between 1995 and 2006, survival of preterm infants, born between 22 and 25 weeks’ gestation, improved by as much as 13% [7]. Similar findings of improved survival have been reported among infants born with extremely low weight (501 to 1500 grams) [2]. Though the main focus has been on low birth weight, several studies have recently also reported U-shaped associations, with increased mortality risks related to high birth weight [1;4;8;9]. The success of improved survival among individuals born early or with extreme birth weight faces challenges such as increased morbidity in infancy and childhood. Long-term survival may also be compromised but few studies have had sufficient length of follow-up to address this issue.

In the present study we took advantage of the unique population-based registries in Denmark to study the entire population of live-born children over a period of 30 years. We investigated how far into adulthood a child’s birth weight influences survival and studied the underlying cause of death.

## Material and Methods

All children born in Denmark are assigned a civil personal registration number allowing for identity-secure linkage of information between national registries. We used data from the Civil Registration System (CRS) [10] to identify all live-born singletons in Denmark between January 1, 1979 and December 31, 2011, with a Danish-born mother (N = 1 708 714). Deaths and dates of death were also identified in the CRS whereas information on the causes of death was extracted from the Cause of Death Registry [11]. Information on birth characteristics e.g. birth weight and gestational age at time of birth were retrieved from the Medical Birth Registry (MBR) [12].

Unless otherwise specified, relative weight was defined as small, large and normal weight for gestational age. Infants born below the 10^th^ percentile birth weight among all infants within the same gestational week, with same gender and born within the same birth cohort were categorized as small for gestational age (SGA). Infants born above the 90^th^ percentile birth weight among all infants within the same gestational week, with same gender and born within the same birth cohort were categorized as large for gestational age (LGA). Infants born within the 11–89% cut-off were categorized as ‘normal for gestational age’ and served as the reference group in most of the analyses.

The relative weight categories were calculated within birth cohort year (1979–1982, 1983–1988, 1989–1994, 1995–2000, 2001–2006, and 2007–2011), gender and gestational age strata. Gestational age at birth was categorised as follows: 19–28 weeks, 29–31 weeks, 32–33 weeks, 34–35 weeks, 36 weeks, 37 weeks, 38–42 weeks, and 43–45 weeks, using mother’s information on first day of last menstruation and sonogram measurements. Only singletons with known gestational age between 19 and 45 gestational weeks and known birth weight between 500 and 7000 grams were included in the study. We compressed coding in the cause of death registry into ten groups using the International Classification of Diseases (ICD) 10^th^ Edition. The ten groups were; infection (ICD-10 code: A00-A09, A15-A99, B00-B99); malignant neoplasm (ICD-10 code: C00-D09); heart disease (ICD-10 code: I00-I25, I27, I30-I52); respiratory disease (ICD-10 code: J00-J99); digestive disease (ICD-10 code: K00-K93); congenital malformation, deformations and chromosomal abnormalities (ICD-10 code: Q00-Q99); condition originating in the perinatal period (ICD-10 code: P00-P96); accident, suicide, and homicide (ICD-10 code: V01-Y89); other; and unknown.

### Statistical analyses

Cox proportional hazards regression was used to estimate hazard ratios [HRs] and 95% confidence intervals [CIs] for the association between small and large gestation weight and mortality over time, using attained age (1-day intervals) as the underlying time scale in all analyses. The baseline hazard function was stratified on gender and birth year. The assumption of proportional hazards was explored by use of cumulative residuals as described by Lin et al [13]. In case of non-proportional hazards, a figure of the HR as a continuous smooth function of age was estimated with natural cubic spline.

Individuals were considered at risk as long as they were registered as living in Denmark. Individuals who emigrated and later immigrated were censored at emigration and reincluded in the cohort at date of immigration to Denmark. To evaluate mortality by years of age, we defined age groups as follows: <2 years, 2–5 years, 6–13 years, 14–19 years, 20–29 years, and 30 years and older. The association between age and relative weight was estimated in each age group after excluding earlier deaths. We also considered the percentile of the relative birth weight as a continuous variable. The effect of this percentile was estimated with a cubic spline plot with knots specified at the 5^th^, 10^th^, 20^th^, 40^th^, 60^th^ and 80^th^ percentile, with the 50^th^ percentile as reference. Cause-specific probabilities of death were estimated non-parametrically with the Aalen-Johansen estimator [14].

All analyses were conducted using SAS 9.4 (SAS Institute Inc., Cary, North Carolina).

### Ethical Considerations

The study was register-based and complied with the regulations and instructions set up by the Danish Data Protection Agency (Danish Protection Board approval no. 2008-54-0472). We only used anonymized data, we only present data in aggregate and anonymous form, and we neither contacted any study participants nor required any active participation from them.

## Results

Overall, 877 303 men and 831 411 women contributed at least one day of observation time between January 1, 1979, and December 31, 2011. Subjects were followed for 27 million person-years and 14 167 deaths occurred in the study period (5 474 in women, 8 693 in men).

The median birth weight changed over calendar time with individuals born between 1979 and 1982 having a median birth weight of 3410 grams and individuals born between 2007 and 2011 a median birth weight of 3550 grams (Table 1). Though birth weight has increased over the past 40 years, the increase has levelled out in most recent years.

**Table 1:** Infant Baseline Characteristics 1979–2011.

		Total Cohort Population (percentile)					
Birth cohort	Characteristics	5^th^	10^th^	50^th^	90^th^	95^th^	Total N
**1979–1982**	**Birth Weight** *	2500	2750	3410	4060	4250	171 782
	**Gestational Age** **	37	38	40	41	42	
**1983–1988**	**Birth Weight** *	2500	2770	3450	4100	4300	295 159
	**Gestational Age** **	37	38	40	41	42	
**1989–1994**	**Birth Weight** *	2580	2820	3500	4150	4350	342 287
	**Gestational Age** **	37	38	40	41	42	
**1995–2000**	**Birth Weight** *	2620	2890	3560	4226	4430	334 109
	**Gestational Age** **	37	38	40	42	42	
**2001–2006**	**Birth Weight** *	2640	2900	3560	4225	4430	314 106
	**Gestational Age** **	37	38	40	42	42	
**2007–2011**	**Birth Weight** *	2640	2880	3550	4195	4390	251 271
	**Gestational Age** **	37	38	40	42	42	

*grams

**weeks

In the figures of cumulated residuals (not show) we observed violations of the proportional hazard assumption in the <2 years old, but no signs of model violations after the age of 2 years. Among SGA individuals, an up to 4.5 fold increased HR was found in the first days of life followed by a strong decline to a relative stable level of about 2.0–2.5 in the age span from 90 days to 2 years, S1 Fig. Among LGA individuals, we observed decreased mortality (although fluctuating) in age <1.5 years, followed by a slightly (non-significant) increased risk in 1.5 to 2 years of age.

**Fig 1:**
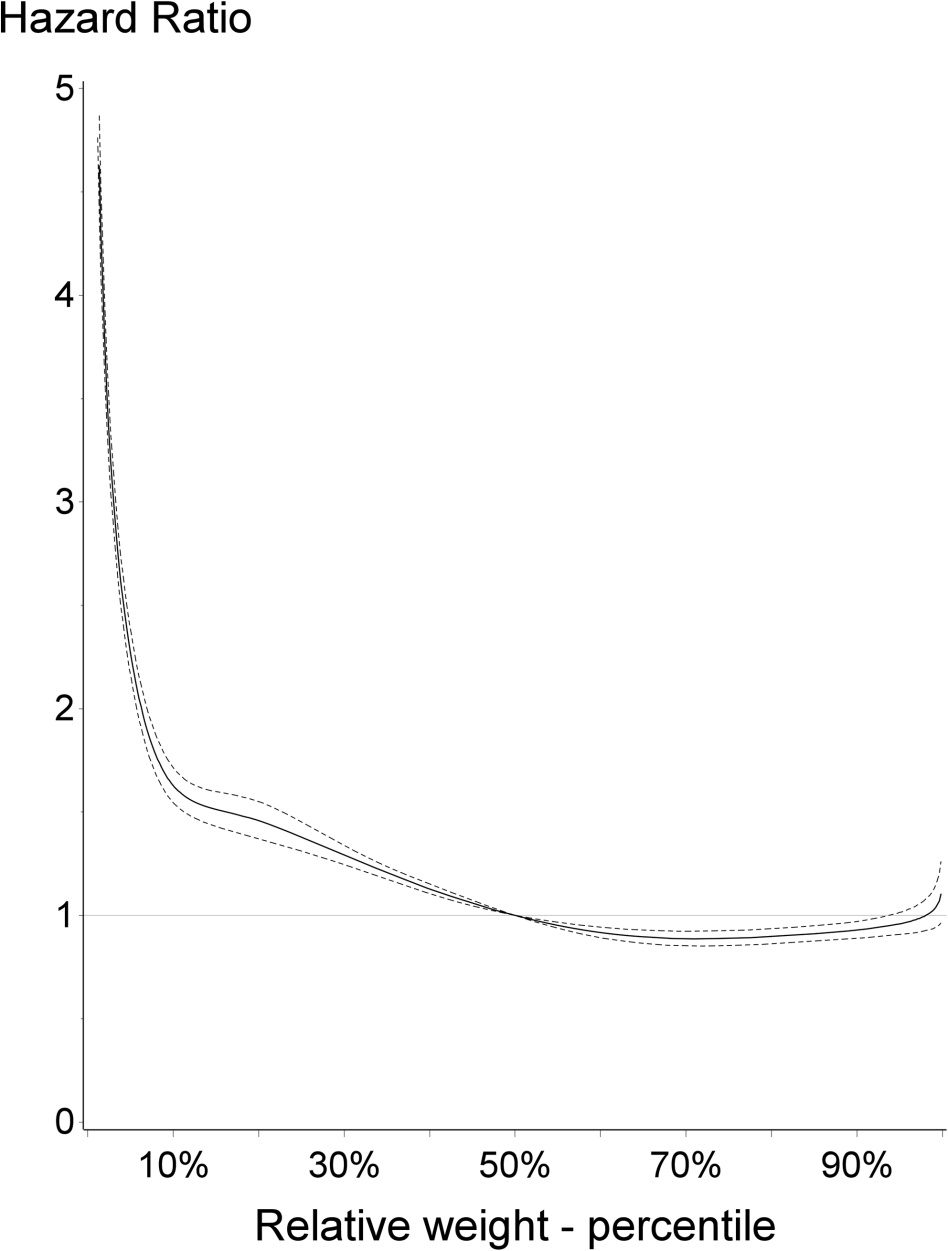
Overall mortality risk by relative birth weight, time period 1979–2011. Hazard ratio and 95% confidence interval presented with 50^th^ percentile as the reference group.

In Fig 1, the overall mortality risk by relative birth weight is presented for the entire study period. Subjects born below the 50^th^ percentile were associated with increased risk of dying, compared with individuals born with normal weight. Conversely, individuals born within the higher percentiles, above the 50^th^ percentile, were at modest decreased risk of dying, compared with individuals born with normal weight for gestation.

Among individuals born with extremely low birth weight, i.e. 500–1499 grams in weeks 19–28, the risk of dying dropped by more than 50% between 1979–1989 and 2000–2011, (S2 and S4 Figs). Among those born weighing 1500–1999 grams in week 36 and onwards, the cumulative mortality risk did not change much over the past 30 years (S2–S4 Figs).

As given in Table 2, individuals small for gestational age were at increased risk of dying throughout young adulthood; HR in ages <2 years: 3.47; 2–5 years 1.70; 6–13 years 1.42; 14–19 years 1.34 and in 20–29 years 1.30. Individuals large for gestational age had a significantly lower mortality risk in the age group <2 years (HR 0.78), whereas no significant association was found in older age groups. Aggregated data for reproducing Table 2, is available as supporting information, S1 Aggregated Data.

**Table 2:** Mortality and Relative Birth Weight by Age Group 1979–2011.

Age group (years)	Relative Birth Weight	Person-Years of Follow-Up (1000s)	No. of Deaths	HR (95% CI)
<2	SGA	318	2349	3.47 (3.30–3.64)
	Normal	2611	5732	1 [Reference]
	LGA	363	601	0.78 (0.72–0.85)
2–5	SGA	588	196	1.70 (1.45–1.99)
	Normal	4706	866	1 [Reference]
	LGA	651	108	0.96 (0.79–1.17)
6–13	SGA	993	163	1.42 (1.20–1.68)
	Normal	7422	813	1 [Reference]
	LGA	974	113	1.14 (0.93–1.38)
14–19	SGA	556	230	1.34 (1.16–1.54)
	Normal	3773	1133	1 [Reference]
	LGA	432	122	0.98 (0.82–1.19)
20–29	SGA	458	264	1.30 (1.14–1.49)
	Normal	2823	1249	1 [Reference]
	LGA	284	132	1.05 (0.88–1.26)
≥30	SGA	23	14	1.06 (0.60–1.88)
	Normal	139	77	1 [Reference]
	LGA	13	5	0.70 (0.28–1.72)

Abbreviation: SGA, small for gestational age; LGA, large for gestational age; HR, Hazard ratio; CI, Confidence interval

When looking at mortality risk by birth cohort in strata of age groups and with one overall reference group (Table 3), survival has improved over time in normal individuals born in years 2007–2011: HR 0.22 (95% CI, 0.19–0.25) compared to individuals born in years 1979–1982. Similarly, in absolute terms, compared to same reference group, survival improved among SGA individuals within the same time span; HR 2.68 (95% CI, 2.37–3.02) in years 1979–1982 to HR 1.28 (95% CI 1.09–1.50) in years 2007–2011. Thereupon, the relative increased mortality among SGA individuals compared to normal born in the period 2007–2011 was almost 6-fold. Increased mortality was also observed among adults (20–29 years), from 1.2-fold increased risk in years 1979–1982 to 1.6-fold increased risk in years 1989–1994.

**Table 3:** Mortality and Relative Birth Weight by Age and Birth Cohort Among Individuals Born Between 1979 and 2011.

		Birth cohort (years)					
		1979–1982	1983–1988	1989–1994	1995–2000	2001–2006	2007–2011
Age group (years)	Relative Birth Weight	HR (95% CI)					
<2	SGA	2.68 (2.37–3.02)	2.38 (2.14–2.64)	2.15 (1.93–2.40)	1.94 (1.73–2.18)	1.96 (1.74–2.21)	1.28 (1.09–1.50)
	Normal	1 [Reference]	0.93 (0.85–1.00)	0.71 (0.65–0.77)	0.46 (0.42–0.50)	0.31 (0.28–0.34)	0.22 (0.19–0.25)
	LGA	0.71 (0.55–0.91)	0.73 (0.61–0.88)	0.52 (0.44–0.62)	0.40 (0.34–0.48)	0.22 (0.18–0.28)	0.21 (0.16–0.29)
2–5	SGA	1.38 (0.95–1.99)	1.60 (1.19–2.14)	0.99 (0.70–1.41)	0.91 (0.61–1.36)	1.02 (0.67–1.54)	0.67 (0.25–1.82)
	Normal	1 [Reference]	0.94 (0.77–1.16)	0.66 (0.53–0.81)	0.53 (0.42–0.67)	0.35 (0.27–0.45)	0.27 (0.16–0.44)
	LGA	0.70 (0.37–1.32)	0.85 (0.55–1.32)	0.75 (0.51–1.11)	0.52 (0.34–0.79)	0.29 (0.17–0.51)	0.50 (0.20–1.21)
6–13	SGA	1.25 (0.87–1.78)	0.99 (0.71–1.37)	1.10 (0.80–1.50)	0.73 (0.48–1.12)	0.71 (0.31–1.60)	
	Normal	1 [Reference]	0.90 (0.75–1.09)	0.55 (0.45–0.68)	0.46 (0.37–0.57)	0.35 (0.24–0.51)	
	LGA	0.90 (0.53–1.53)	1.27 (0.90–1.79)	0.74 (0.51–1.07)	0.38 (0.24–0.61)	0.26 (0.10–0.70)	
14–19	SGA	1.13 (0.85–1.49)	1.11 (0.88–1.40)	1.12 (0.87–1.44)	NA		
	Normal	1 [Reference]	0.82 (0.71–0.94)	0.68 (0.58–0.79)	0.49 (0.32–0.75)		
	LGA	1.07 (0.75–1.55)	0.90 (0.67–1.20)	0.57 (0.41–0.80)	0.13 (0.02–0.92)		
20–29	SGA	1.20 (0.99–1.46)	1.20 (0.99–1.47)	1.61 (1.04–2.51)			
	Normal	1 [Reference]	0.90 (0.80–1.02)	0.73 (0.56–0.95)			
	LGA	1.00 (0.77–1.32)	1.05 (0.82–1.35)	0.45 (0.19–1.09)			
>30	SGA	1.06 (0.60–1.87)					
	Normal	1 [Reference]					
	LGA	0.69 (0.28–1.72)					

Abbreviation: SGA, small for gestational age; LGA, large for gestational age; HR, Hazard ratio; CI, Confidence interval; NA, not applicable

The cumulative mortality by any cause of death was estimated using the Aalen-Johansen estimator between years 1979 and 2011. Risk of death stratified by relative weight (SGA, Normal, LGA) and by disease group is shown in more detail, S5 Fig. Briefly, before 1 year of age SGA individuals were at 1.5% increased risk of dying, while the mortality risks among normal and LGA were much lower, 0.41% and 0.42% respectively. Besides the first year of life being the highest risk of dying, risks were still increasing among SGA individuals up to age 30 years, 2.8%. Among normal and LGA individuals the corresponding risks were 1.4% and 1.3% respectively.

Individuals born SGA were at increased risk of dying, in all disease categories but malignant neoplasms, Table 4. The strongest risks were observed for congenital malformations (HR, 3.63; 95% CI, 3.40–3.87) and perinatal conditions (HR, 3.44; 95% CI, 3.20–3.70). Statistically significant increased risks were also observed for dying from respiratory disease, heart diseases and digestive diseases, Table 4. Individuals born LGA were at decreased risk of dying from congenital malformations and perinatal conditions (HR, 0.90; 95% CI, 0.79–1.02 vs. HR, 0.64; 95% CI, 0.55–0.74). Noteworthy, increased risk of dying from malignant neoplasms was observed among LGA individuals, HR, 1.21; 95% CI, 1.01–1.46.

**Table 4:** Mortality by Relative Gestational Weight and Cause of Death 1979–2011.

Cause of Death	Relative Birth Weight*	No. of Deaths	HR (95% CI)
Infection	SGA	133	1.52 (1.22–1.90)
	Normal	329	1 [Reference]
	LGA	24	0.74 (0.48–1.12)
Malignant Neoplasm	SGA	408	0.99 (0.88–1.12)
	Normal	1277	1 [Reference]
	LGA	128	1.21 (1.01–1.46)
Heart Disease	SGA	113	1.56 (1.21–2.02)
	Normal	189	1 [Reference]
	LGA	21	1.27 (0.80–2.00)
Respiratory Disease	SGA	178	1.84 (1.51–2.25)
	Normal	327	1 [Reference]
	LGA	32	1.05 (0.73–1.52)
Digestive Disease	SGA	80	1.56 (1.15–2.11)
	Normal	141	1 [Reference]
	LGA	14	1.18 (0.68–2.05)
Congenital malformation	SGA	1839	3.63 (3.40–3.87)
	Normal	2488	1 [Reference]
	LGA	260	0.90 (0.79–1.02)
Perinatal Condition	SGA	1729	3.44 (3.20–3.70)
	Normal	2443	1 [Reference]
	LGA	197	0.64 (0.55–0.74)
Accident, Suicide, Homicide	SGA	1787	1.21 (1.14–1.29)
	Normal	3711	1 [Reference]
	LGA	307	1.00 (0.89–1.12)
Other	SGA	1245	1.72 (1.60–1.84)
	Normal	2864	1 [Reference]
	LGA	230	0.79 (0.69–0.90)
Unknown	SGA	77	1.42 (1.06–1.89)
	Normal	202	1 [Reference]
	LGA	14	0.62 (0.36–1.07)

Abbreviation: SGA, small for gestational age; LGA, large for gestational age; HR, Hazard ratio; CI, Confidence interval

*Person-Years of Follow-Up (1000s): SGA, 7546; Normal, 29137; LGA, 2929

When we modelled the effect of relative weight as a function of age stratified by cause of death, SGA born individuals were at strongest increased risk of dying from infection; heart disease; respiratory disease; perinatal conditions; congenital malformation; accidents, suicide, and homicide during the first years of life. LGA-born individuals had a decreased risk of dying from congenital malformations and perinatal condition during the first years of life. Further, in the LGA-born individuals, the risk of dying from malignant neoplasms was highest between age 6 and 13 years, (HR, 1.56; 95% CI, 1.13–2.16). Increased risk of dying from digestive disease (HR, 6.80; 95% CI, 2.11–21.91), (2–5 years old) and heart disease (HR, 2.37; 95% CI, 1.04–5.44), (14–19 years old) was also observed, S1 Table.

## Discussion

Our study observed high mortality among individuals born small for gestational age between years 1979 and 2011. With a total of 1.7 million live-born children followed for 27 million person-years during a 30-year period, our study had unique power to describe the long-term survival among individuals born small and large for gestational age. This also allowed us to investigate how mortality decreased over calendar time independently of birth weight. In the USA reports have shown that prenatal and neonatal care improved during the 90’s [2]. Noteworthy, the change in perinatal and neonatal care can be explained by increased use of caesarean section, steroid treatment, and respiratory aid. The North American study reported declining mortality among infants born with a birth weight below 1500 grams until 1995, but no further decline at end of follow-up in 1999. In the present study, we observed a steady decline in mortality over time among children in all relative weight groups, all the way up to the end of follow-up in 2011.

We are not aware of any other study that has reported on the association between being born small or large for gestational age and mortality risk up to 30 years after birth. Previous studies on this topic have primarily explored the association of birth weight and gestational age separately. As an example a study based on the Swedish medical birth registry reported increased mortality in infancy, early childhood and young adulthood among individuals born at 37–38 weeks of gestation [15]. In the present study we took this a step further and combined gestational age with weight. It is well known that SGA infants are at higher mortality risk than non-SGA infants or infants born within the normal weight span [16;17]. We found this increased mortality risk to be present in all strata of year of birth and age. The mortality risks among SGA individuals were 4.5-folded the first days of life, with a strong reduced risk the following 90 days, and further decreased up to adulthood. The increased mortality in early age may be expected; but the elevated mortality up to adulthood however, was somewhat surprising.

Mortality risks decreased over calendar time, but were still strong within each birth cohort among individuals born small for gestational age. Interestingly, though advanced neonatal care and improved survival of SGA individuals likely result in different long-term outcome for individuals born in the 1970’s and 2011, we observed similar increased risks in comparing SGA individuals to normal individuals with the highest difference in the later time period. A possible explanation to this could be that care of those born extremely early and/or extremely small have improved, consequently more extreme individuals survive their first day in life and are therefore more frequently included in the later cohorts. Thereby, one can argue that relative weight is a rough estimate, suggesting that the definition of SGA should be reconsidered.

In general, SGA born individuals require more intervention, longer stay at hospital with neonatal care and are at risk for certain conditions compared to individuals born normal weight for gestational age. Preterm SGA and term SGA born individuals face different medical conditions. Previous report discovered that term born SGA individuals are likely related to sociodemographic status while preterm are related to chronic hypertension and preeclampsia [18]. Also discovered in the same study, term SGA individuals are more likely to face prenatal deaths while preterm neonatal death. In the present study we do not differ between preterm and term SGA individuals. With regard to that our study is restricted to only live born individuals, it is possible that our findings corresponds to SGA individuals born preterm rather than term.

In a previous study the authors observed a 2-fold increased risk of prenatal and neonatal death among term and post–term individuals in the 15^th^ birth weight percentile, compared with the previously used 10^th^ percentile [9]. In our study we observed a 2-fold increased mortality risk below the 7.5^th^ percentile. We took into account birth cohort, gender and gestational age when setting the cut-offs for relative birth weight, and this difference in study design and study population could explain the dissimilarities comparing our results with earlier published results.

Some studies have reported an U-shaped association between birth weight and all-cause mortality [1]. Overall we observed a decrease in mortality risk within each birth cohort among individuals born large for gestational age, with the strongest effect in infants and toddlers. This contrasts with earlier studies observing an increased risk or no significant risk difference between LGA and normal individuals [9;16]. It is however possible that our study including individuals below 40 years of age, is too young to show metabolic and cardiovascular consequences of LGA. An earlier Norwegian study investigated survival among post-term infants. The authors found post-term infants to be at increased risk of dying before one year of age. Further, among boys, increased risks were observed also in late childhood [8]. We have no immediate explanation for these differences in our findings. However when investigating cause-specific mortality, we observed that malignant neoplasms was associated with increased mortality risk among LGA individuals; this partly confirms earlier findings of increased cancer mortality among adults per kg increased birth weight [4].

The major strengths of the present study are the prospective study design, the long follow-up, and the large sample size. The registry-based design allowed us to assemble a nationwide cohort with independent ascertainment of exposure and outcome and complete follow-up of all infants with accurate longitudinal information. In Denmark health care is free of charge and encouraged for all pregnant women irrespective of income and immigrant status. We used the Danish CRS [10;19] and thereby covered complete information on maternal identity among women born in Denmark in April 1935 or later and all their children born in Denmark in 1960 or later. Full information on immigrations, emigrations and permanent residence by municipality and full address in Denmark was covered from 1977 and onwards [19]. There are a few drawbacks with the Danish MBR [12]. In the years 1973–1978 the weights were reported rounded by 500 gram [20]; to avoid inducing non-differential misclassification we excluded information from these years. The use of digital scales replaced the mechanical scales in 1996 and thereafter weight was no longer presented by 50- and 100-gram values. The possible impact on the calendar time analysis was reduced by restricting the classification of the overall relative weight to the years 1979 to 2011. Further, during 1973–79 gestational age was coded in intervals with regard to the number of weeks counted as preterm. In the following years gestational age was registered as weeks or days. To unify the coding we therefore used early registered gestational age categories for the entire calendar time in our classification of gestational age. Further we had no possibility to distinguish between gestational age measures (last menstrual period or ultrasound dates), but believe this has had little impact on our study. A few problems regarding data in the Cause of Death Registry [11] have been observed. In most of our analyses we used all-cause of death where possible misclassification or discontinuity in registrations would have no impact on the results.

## Conclusion

In conclusion, our findings suggest that weight by gestational age at birth is an important predictor for mortality from birth until approximately at least 30 years of age. Multiple causes of death were associated with being born small for gestational age during this age span.

## Supporting Information

S1 Aggregated Data (ZIP)

S1 Fig Mortality by Relative Gestational Weight in 0–720 days old, as a continues function of time, 1979–2011. (PDF)

S2 Fig Cumulative Mortality Risk by Birth Weight Group, Gestational Age, Age at Death at Birth Cohort 1979–1989. (PDF)

S3 Fig Cumulative Mortality Risk by Birth Weight Group, Gestational Age, Age at Death at Birth Cohort 1990–1999. (PDF)

S4 Fig Cumulative Mortality Risk by Birth Weight Group, Gestational Age, Age at Death at Birth Cohort 2000–2011. (PDF)

S5 Fig Cumulative Mortality, by Age, Cause of Death Stratified by Relative Birth Weight at 1979–2011. (PDF)

S1 Table Mortality by Relative Gestational Weight, Cause of Death and Age Group 1979–2011 (DOC)

## Abbreviations

CI: confidence interval
CRS: Civil Registration System
HR: hazard ratio
ICD: International Classification of Diseases
LGA: large for gestation age
MBR: Medical Birth Registry
SGA: small for gestational age
