# Supplementary figures and images for: Long-Term Survival of Individuals Born Small and Large for Gestational Age
Source: PLoS One. 2015 Sep 21;10(9):e0138594. doi: 10.1371/journal.pone.0138594 (PMC4577072; doi:10.1371/journal.pone.0138594)

# Hazard Ratio

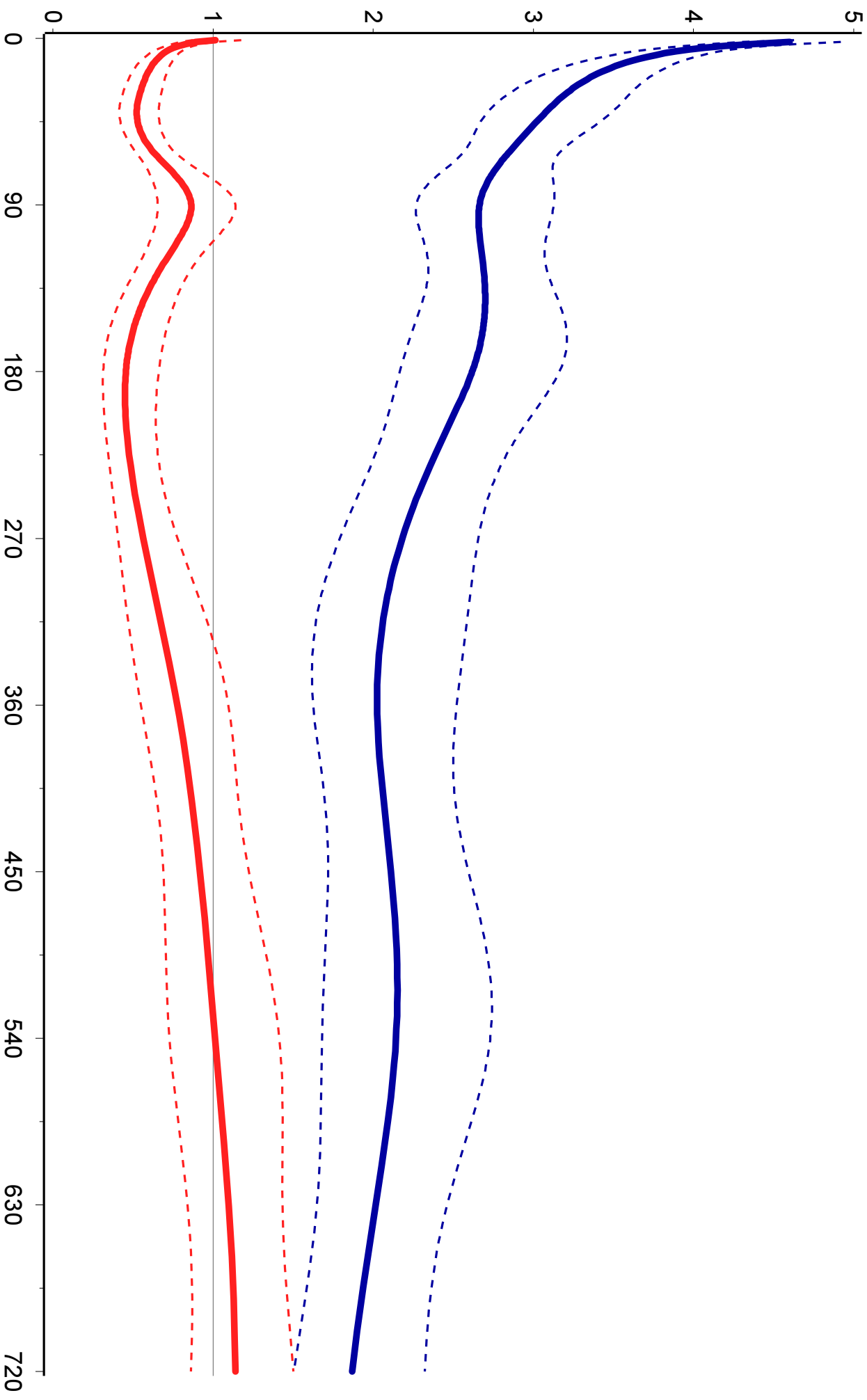

Supplement: S1 Fig — (PDF) [file pone.0138594.s002.pdf]
