# Supplementary material for: Long-Term Survival of Individuals Born Small and Large for Gestational Age
Source: PLoS One. 2015 Sep 21;10(9):e0138594. doi: 10.1371/journal.pone.0138594 (PMC4577072; doi:10.1371/journal.pone.0138594)

Birth year: 1979-1989

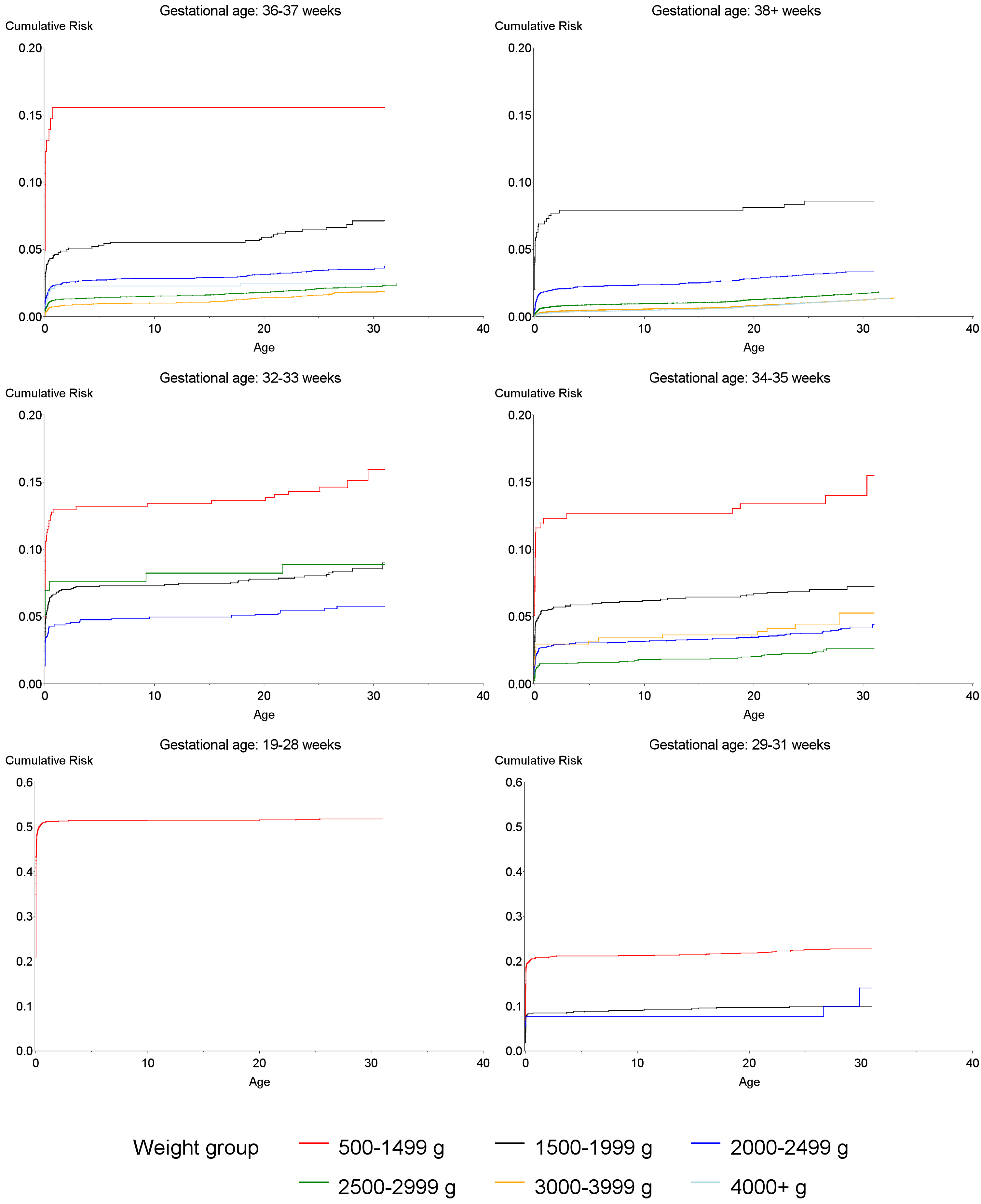

Supplement: S2 Fig — (PDF) [file pone.0138594.s003.pdf]
