# Supplementary material for: Long-Term Survival of Individuals Born Small and Large for Gestational Age
Source: PLoS One. 2015 Sep 21;10(9):e0138594. doi: 10.1371/journal.pone.0138594 (PMC4577072; doi:10.1371/journal.pone.0138594)
Supplement: S5 Fig — (PDF) [file pone.0138594.s006.pdf]

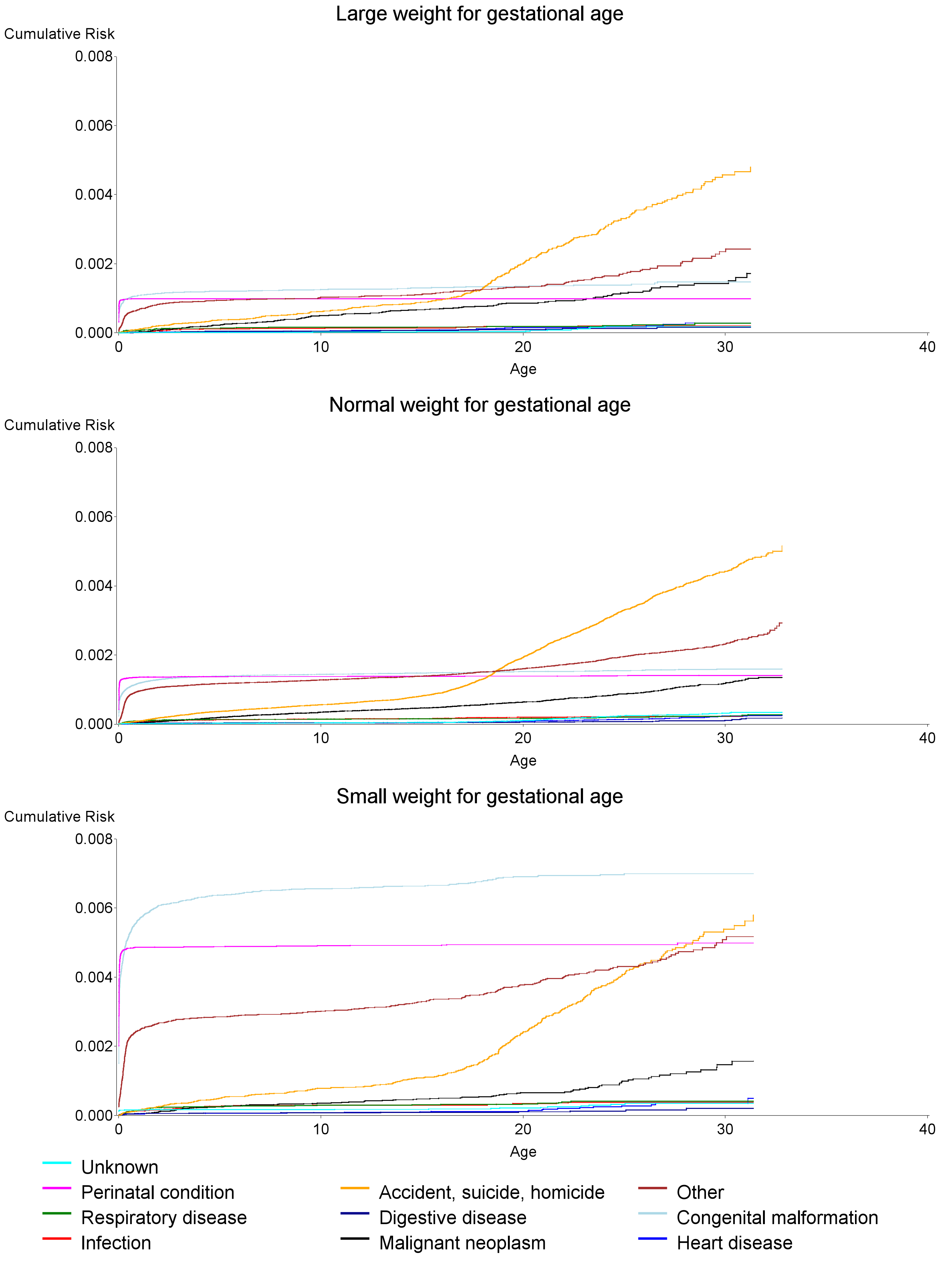

Large weight for gestational age
Cumulative Risk
0.008
0.006
0.004
0.002
0.000
0
10
20
30
40
Age
Normal weight for gestational age
Cumulative Risk
0.008
0.006
0.004
0.002
0.000
0
10
20
30
40
Age
Small weight for gestational age
Cumulative Risk
0.008
0.006
0.004
0.002
0.000
0
10
20
30
40
Age
Unknown
Perinatal condition
Respiratory disease
Infection
Accident, suicide, homicide
Digestive disease
Malignant neoplasm
Other
Congenital malformation
Heart disease
