# Supplementary material for: Long-Term Survival of Individuals Born Small and Large for Gestational Age
Source: PLoS One. 2015 Sep 21;10(9):e0138594. doi: 10.1371/journal.pone.0138594 (PMC4577072; doi:10.1371/journal.pone.0138594)
Supplement: S1 Table — (DOC) [file pone.0138594.s007.doc]

| **S1 Table. Mortality by Relative Gestational Weight, Cause of Death and Age Group, 1979-2011** | | | | | |
| --- | --- | --- | --- | --- | --- |
| **Cause of Death** | **Age group (years)** | **Relative Birth Weight** | **Person-Years of Follow-Up (1000s)** | **No. of Deaths** | **HR (95% CI)** |
| **Infection** | <2 | SGA | 578 | 63 | 2.03 (1.47-2.81) |
|  |  | Normal | 3044 | 146 | 1 [Reference] |
|  |  | LGA | 374 | 6 | 0.36 (0.16-0.82) |
|  | 2-5 | SGA | 1105 | 30 | 1.72 (1.09-2.71) |
|  |  | Normal | 5566 | 77 | 1 [Reference] |
|  |  | LGA | 675 | 10 | 1.22 (0.63-2.37) |
|  | 6-13 | SGA | 2022 | 11 | 0.72 (0.35-1.48) |
|  |  | Normal | 9136 | 39 | 1 [Reference] |
|  |  | LGA | 1021 | 3 | 1.06 (0.32-3.46) |
|  | 14-19 | SGA | 1325 | 16 | 1.23 (0.66-2.29) |
|  |  | Normal | 5052 | 42 | 1 [Reference] |
|  |  | LGA | 467 | 4 | 1.22 (0.43-3.44) |
|  | 20-29 | SGA | 1714 | 8 | 0.87 (0.35-2.12) |
|  |  | Normal | 4913 | 18 | 1 [Reference] |
|  |  | LGA | 341 | 1 | 0.78 (0.10-5.93) |
|  | ≥30 | SGA | 799 | 5 | 1.21 (0.35-4.16) |
|  |  | Normal | 1423 | 7 | 1 [Reference] |
|  |  | LGA |  | 0 | 0 |
| **Malignant Neoplasm** | <2 | SGA | 578 | 32 | 1.07 (0.71-1.62) |
|  |  | Normal | 3044 | 131 | 1 [Reference] |
|  |  | LGA | 374 | 16 | 1.16 (0.69-1.96) |
|  | 2-5 | SGA | 1105 | 51 | 0.96 (0.70-1.33) |
|  |  | Normal | 5566 | 236 | 1 [Reference] |
|  |  | LGA | 675 | 30 | 1.16 (0.79-1.70) |
|  | 6-13 | SGA | 2022 | 77 | 0.90 (0.68-1.18) |
|  |  | Normal | 9136 | 285 | 1 [Reference] |
|  |  | LGA | 1021 | 43 | 1.56 (1.13-2.16) |
|  | 14-19 | SGA | 1325 | 55 | 1.14 (0.82-1.57) |
|  |  | Normal | 5052 | 173 | 1 [Reference] |
|  |  | LGA | 467 | 11 | 0.74 (0.40-1.36) |
|  | 20-29 | SGA | 1714 | 97 | 0.95 (0.74-1.22) |
|  |  | Normal | 4913 | 279 | 1 [Reference] |
|  |  | LGA | 341 | 21 | 1.13 (0.72-1.77) |
|  | ≥30 | SGA | 799 | 96 | 1.04 (0.80-1.35) |
|  |  | Normal | 1423 | 173 | 1 [Reference] |
|  |  | LGA | 47 | 7 | 1.38 (0.64-2.96) |
| Abbreviation: SGA, small for gestational age; LGA, large for gestational age; HR, Hazard ratio; CI, Confidence interval | | | | | |

| **S1 Table. Mortality by Relative Gestational Weight, Cause of Death and Age Group, 1979-2011 (continued)** | | | | | |
| --- | --- | --- | --- | --- | --- |
| **Cause of Death** | **Age group (years)** | **Relative Birth Weight** | **Person-Years of Follow-Up (1000s)** | **No. of Deaths** | **HR (95% CI)** |
| **Heart Disease** | <2 | SGA | 578 | 21 | 2.80 (1.57-5.01) |
|  |  | Normal | 3044 | 37 | 1 [Reference] |
|  |  | LGA | 374 | 4 | 0.91 (0.32-2.56) |
|  | 2-5 | SGA | 1105 | 4 | 2.67 (0.78-9.18) |
|  |  | Normal | 5566 | 9 | 1 [Reference] |
|  |  | LGA | 675 | 3 | 2.74 (0.74-10.23) |
|  | 6-13 | SGA | 2022 | 4 | 1.00 (0.31-3.23) |
|  |  | Normal | 9136 | 17 | 1 [Reference] |
|  |  | LGA | 1021 | 3 | 1.50 (0.43-5.16) |
|  | 14-19 | SGA | 1325 | 6 | 0.75 (0.30-1.90) |
|  |  | Normal | 5052 | 31 | 1 [Reference] |
|  |  | LGA | 467 | 7 | 2.37 (1.04-5.44) |
|  | 20-29 | SGA | 1714 | 38 | 1.59 (1.02-2.50) |
|  |  | Normal | 4913 | 59 | 1 [Reference] |
|  |  | LGA | 341 | 3 | 0.67 (0.21-2.13) |
|  | ≥30 | SGA | 799 | 40 | 1.34 (0.84-2.14) |
|  |  | Normal | 1423 | 36 | 1 [Reference] |
|  |  | LGA | 47 | 1 | 0.73 (0.10-5.38) |
| **Respiratory Disease** | <2 | SGA | 578 | 109 | 1.97 (1.53-2.55) |
|  |  | Normal | 3044 | 205 | 1 [Reference] |
|  |  | LGA | 374 | 22 | 1.08 (0.70-1.69) |
|  | 2-5 | SGA | 1105 | 21 | 1.82 (1.02-3.23) |
|  |  | Normal | 5566 | 40 | 1 [Reference] |
|  |  | LGA | 675 | 5 | 1.36 (0.53-3.49) |
|  | 6-13 | SGA | 2022 | 15 | 2.31 (1.11-4.82) |
|  |  | Normal | 9136 | 21 | 1 [Reference] |
|  |  | LGA | 1021 | 1 | 0.48 (0.06-3.64) |
|  | 14-19 | SGA | 1325 | 7 | 1.35 (0.50-3.61) |
|  |  | Normal | 5052 | 15 | 1 [Reference] |
|  |  | LGA | 467 | 1 | 0.76 (0.10-5.79) |
|  | 20-29 | SGA | 1714 | 11 | 1.07 (0.51-2.24) |
|  |  | Normal | 4913 | 31 | 1 [Reference] |
|  |  | LGA | 341 | 3 | 1.23 (0.37-4.07) |
|  | ≥30 | SGA | 799 | 15 | 1.83 (0.85-3.94) |
|  |  | Normal | 1423 | 15 | 1 [Reference] |
|  |  | LGA | NA | 0 | 0 |
| Abbreviation: SGA, small for gestational age; LGA, large for gestational age; HR, Hazard ratio; CI, Confidence interval | | | | | |

| **S1 Table. Mortality by Relative Gestational Weight, Cause of Death and Age Group, 1979-2011 (continued)** | | | | | |
| --- | --- | --- | --- | --- | --- |
| **Cause of Death** | **Age group (years)** | **Relative Birth Weight** | **Person-Years of Follow-Up (1000s)** | **No. of Deaths** | **HR (95% CI)** |
| **Digestive Disease** | <2 | SGA | 578 | 28 | 1.54 (0.93-2.55) |
|  |  | Normal | 3044 | 64 | 1 [Reference] |
|  |  | LGA | 374 | 2 | 0.30 (0.07-1.25) |
|  | 2-5 | SGA | 1105 | 4 | 2.14 (0.56-8.16) |
|  |  | Normal | 5566 | 7 | 1 [Reference] |
|  |  | LGA | 675 | 5 | 6.80 (2.11-21.91) |
|  | 6-13 | SGA | 2022 | 4 | 2.04 (0.53-7.90) |
|  |  | Normal | 9136 | 7 | 1 [Reference] |
|  |  | LGA | 1021 | 2 | 2.64 (0.54-13.00) |
|  | 14-19 | SGA | 1325 | 3 | 1.37 (0.36-5.17) |
|  |  | Normal | 5052 | 11 | 1 [Reference] |
|  |  | LGA | 467 | 2 | 2.19 (0.48-10.04) |
|  | 20-29 | SGA | 1714 | 17 | 1.90 (0.96-3.74) |
|  |  | Normal | 4913 | 24 | 1 [Reference] |
|  |  | LGA | 341 | 3 | 1.77 (0.53-6.00) |
|  | ≥30 | SGA | 799 | 24 | 1.25 (0.70-2.24) |
|  |  | Normal | 1423 | 28 | 1 [Reference] |
|  |  | LGA | NA | 0 | 0 |
| **Congenital Malformation** | <2 | SGA | 578 | 1593 | 3.84 (3.58-4.11) |
|  |  | Normal | 3044 | 2125 | 1 [Reference] |
|  |  | LGA | 374 | 229 | 0.91 (0.79-1.04) |
|  | 2-5 | SGA | 1105 | 111 | 2.85 (2.19-3.71) |
|  |  | Normal | 5566 | 168 | 1 [Reference] |
|  |  | LGA | 675 | 12 | 0.65 (0.36-1.17) |
|  | 6-13 | SGA | 2022 | 56 | 2.40 (1.68-3.44) |
|  |  | Normal | 9136 | 94 | 1 [Reference] |
|  |  | LGA | 1021 | 7 | 0.75 (0.35-1.62) |
|  | 14-19 | SGA | 1325 | 45 | 3.12 (2.03-4.81) |
|  |  | Normal | 5052 | 52 | 1 [Reference] |
|  |  | LGA | 467 | 6 | 1.33 (0.57-3.12) |
|  | 20-29 | SGA | 1714 | 26 | 1.64 (0.96-2.81) |
|  |  | Normal | 4913 | 40 | 1 [Reference] |
|  |  | LGA | 341 | 6 | 2.33 (0.97-5.61) |
|  | ≥30 | SGA | 799 | 8 | 1.86 (0.68-5.08) |
|  |  | Normal | 1423 | 9 | 1 [Reference] |
|  |  | LGA | NA | 0 | 0 |
| Abbreviation: SGA, small for gestational age; LGA, large for gestational age; HR, Hazard ratio; CI, Confidence interval | | | | | |

| **S1 Table. Mortality by Relative Gestational Weight, Cause of Death and Age Group, 1979-2011 (continued)** | | | | | |
| --- | --- | --- | --- | --- | --- |
| **Cause of Death** | **Age group (years)** | **Relative Birth Weight** | **Person-Years of Follow-Up (1000s)** | **No. of Deaths** | **HR (95% CI)** |
| **Perinatal Condition** | <2 | SGA | 578 | 1711 | 3.46 (3.21-3.72) |
|  |  | Normal | 3 044 | 2403 | 1 [Reference] |
|  |  | LGA | 374 | 197 | 0.65 (0.56-0.75) |
|  | 2-5 | SGA | 105 | 3 | 1.58 (0.46-5.42) |
|  |  | Normal | 5566 | 17 | 1 [Reference] |
|  |  | LGA | NA | 0 | 0 |
|  | 6-13 | SGA | 2022 | 4 | 11.71 (2.58-53.18) |
|  |  | Normal | 9136 | 3 | 1 [Reference] |
|  |  | LGA | NA | 0 | 0 |
|  | 14-19 | SGA | 1325 | 2 | 2.71 (0.52-13.98) |
|  |  | Normal | 5052 | 5 | 1 [Reference] |
|  |  | LGA | NA | 0 | 0 |
|  | 20-29 | SGA | 1714 | 7 | 2.03 (0.82-5.01) |
|  |  | Normal | 4913 | 15 | 1 [Reference] |
|  |  | LGA | NA | 0 | 0 |
|  | ≥30 | SGA | NA | 0 | 0 |
|  |  | Normal | NA | 0 | 0 |
|  |  | LGA | NA | 0 | 0 |
| **Accident, Suicide, Homicide** | <2 | SGA | 578 | 108 | 1.36 (1.07-1.73) |
|  |  | Normal | 3044 | 314 | 1 [Reference] |
|  |  | LGA | 374 | 34 | 1.01 (0.71-1.40) |
|  | 2-5 | SGA | 1105 | 119 | 1.15 (0.92-1.44) |
|  |  | Normal | 5566 | 373 | 1 [Reference] |
|  |  | LGA | 675 | 35 | 0.96 (0.68-1.36) |
|  | 6-13 | SGA | 2022 | 177 | 1.27 (1.05-1.54) |
|  |  | Normal | 9136 | 447 | 1 [Reference] |
|  |  | LGA | 1021 | 48 | 1.17 (0.87-1.58) |
|  | 14-19 | SGA | 1325 | 399 | 1.23 (1.08-1.40) |
|  |  | Normal | 5052 | 974 | 1 [Reference] |
|  |  | LGA | 467 | 86 | 0.99 (0.79-1.24) |
|  | 20-29 | SGA | 1714 | 694 | 1.17 (1.06-1.29) |
|  |  | Normal | 4913 | 1294 | 1 [Reference] |
|  |  | LGA | 341 | 90 | 0.94 (0.75-1.16) |
|  | ≥30 | SGA | 799 | 290 | 1.20 (1.02-1.42) |
|  |  | Normal | 1423 | 309 | 1 [Reference] |
|  |  | LGA | 47 | 14 | 1.07 (0.63-1.84) |
| Abbreviation: SGA, small for gestational age; LGA, large for gestational age; HR, Hazard ratio; CI, Confidence interval | | | | | |

| **S1 Table. Mortality by Relative Gestational Weight, Cause of Death and Age Group,** **1979-2011 (continued)** | | | | | |
| --- | --- | --- | --- | --- | --- |
| **Cause of Death** | **Age group (years)** | **Relative Birth Weight** | **Person-Years of Follow-Up (1000s)** | **No. of Deaths** | **HR (95% CI)** |
| **Other** | <2 | SGA | 578 | 644 | 2.03 (1.85-2.20) |
|  |  | Normal | 3044 | 1664 | 1 [Reference] |
|  |  | LGA | 374 | 143 | 0.75 (0.63-0.88) |
|  | 2-5 | SGA | 1105 | 69 | 1.46 (1.08-1.96) |
|  |  | Normal | 5566 | 206 | 1 [Reference] |
|  |  | LGA | 675 | 21 | 0.93 (0.59-1.46) |
|  | 6-13 | SGA | 2022 | 82 | 1.68 (1.27-2.23) |
|  |  | Normal | 9136 | 198 | 1 [Reference] |
|  |  | LGA | 021 | 18 | 0.86 (0.53-1.40) |
|  | 14-19 | SGA | 1325 | 82 | 1.31 (1.00-1.72) |
|  |  | Normal | 5052 | 231 | 1 [Reference] |
|  |  | LGA | 467 | 15 | 0.74 (0.44-1.25) |
|  | 20-29 | SGA | 1714 | 209 | 1.36 (1.13-1.63) |
|  |  | Normal | 4913 | 393 | 1 [Reference] |
|  |  | LGA | 341 | 26 | 0.99 (0.66-1.47) |
|  | ≥30 | SGA | 799 | 159 | 1.40 (1.11-1.75) |
|  |  | Normal | 1423 | 172 | 1 [Reference] |
|  |  | LGA | 47 | 7 | 1.04 (0.49-2.23) |
| **Unknown** | <2 | SGA | 578 | 27 | 9.44 (5.38-16.55) |
|  |  | Normal | 3044 | 24 | 1 [Reference] |
|  |  | LGA | 374 | 4 | 0.96 (0.33-2.77) |
|  | 2-5 | SGA | 1105 | 3 | 1.77 (0.41-7.62) |
|  |  | Normal | 5566 | 9 | 1 [Reference] |
|  |  | LGA | 675 | 1 | 0.73 (0.09-5.80) |
|  | 6-13 | SGA | 2022 | 2 | 0.67 (0.15-3.01) |
|  |  | Normal | 9136 | 20 | 1 [Reference] |
|  |  | LGA | 1021 | 1 | 0.35 (0.05-2.59) |
|  | 14-19 | SGA | 1325 | 4 | 0.77 (0.27-2.17) |
|  |  | Normal | 5052 | 35 | 1 [Reference] |
|  |  | LGA | 467 | 1 | 0.26 (0.04-1.93) |
|  | 20-29 | SGA | 1714 | 26 | 0.90 (0.56-1.44) |
|  |  | Normal | 4913 | 87 | 1 [Reference] |
|  |  | LGA | 341 | 7 | 0.93 (0.43-2.02) |
|  | ≥30 | SGA | 799 | 15 | 0.73 (0.38-1.40) |
|  |  | Normal | 1423 | 27 | 1 [Reference] |
|  |  | LGA | NA | 0 | 0 |
| Abbreviation: SGA, small for gestational age; LGA, large for gestational age; HR, Hazard ratio; CI, Confidence interval | | | | | |
